# Jianpi Biyan formula ameliorates spleen-deficiency-type allergic rhinitis and modulates the gut–nose axis: Integrated evidence from gut microbiota, SCFA metabolism, and TLR4/NF-κB signaling

**DOI:** 10.3389/fimmu.2026.1922742

**Published:** 2026-09-24

**Authors:** Meihui Tian, Yurong Huang, Weifang Sun, Yanan Zhang, Yong Tang

**Affiliations:** 1 Department of Traditional Chinese Medicine Rehabilitation, the Fourth Affiliated Hospital of School of Medicine, and International School of Medicine, International Institutes of Medicine, Zhejiang University, Yiwu, China; 2 College of Integrated Chinese and Western Medicine, Changchun University of Chinese Medicine, Changchun, China; 3 College of Traditional Chinese Medicine, Changchun University of Chinese Medicine, Changchun, China; 4 Department of Otolaryngology-Head and Neck Surgery, The People’s Hospital of Jilin Province, Changchun, Jilin, China

**Keywords:** allergic rhinitis, gut microbiota, gut-nose axis, Jianpi Biyan formula, TLR4/NF-κB pathway

## Abstract

The Jianpi Biyan formula (JPBY) is a modern Chinese herbal compound derived from the classic Qing Dynasty formula “Jiegeng Yuanshen Decoction.” It is primarily used to treat allergic rhinitis (AR) with spleen deficiency as the core pathogenesis and has demonstrated good clinical efficacy; however, its modern biological mechanisms remain unclear. This study investigated the effects of JPBY on spleen-deficiency-type AR, with particular emphasis on the gut microbiota, short-chain fatty acid (SCFA) metabolism, mucosal barrier integrity, and TLR4/NF-κB-associated inflammatory responses. A rat model of spleen-deficiency-type AR was established to evaluate the effects of JPBY on nasal symptoms, spleen deficiency-related manifestations, gastrointestinal function, and intestinal and nasal mucosal integrity. Gut microbiota profiling, SCFA metabolomics, and transcriptomic analyses were integrated to characterize JPBY-associated changes in gut and nasal inflammatory and metabolic profiles. Serum-absorbed components of JPBY were identified using UHPLC-MS/MS, and network pharmacology, molecular docking, immunofluorescence, Western blotting, and ELISA were subsequently employed to investigate potential molecular targets and signaling pathways. JPBY significantly alleviated nasal symptoms and spleen deficiency-related manifestations, improved gastrointestinal digestive function, and enhanced intestinal and nasal mucosal barrier integrity in spleen-deficiency-type AR rats. JPBY also markedly altered the composition of the gut microbiota and SCFA metabolism. Integrated multi-omics analyses identified associations among gut microbial alterations, SCFA metabolism, mucosal barrier function, and inflammatory signaling. In particular, TLR4/NF-κB signaling was identified as a potential molecular pathway associated with inflammatory changes in both intestinal and nasal tissues. Consistent with these findings, JPBY suppressed TLR4/NF-κB-associated signaling in both colonic and nasal tissues and modulated the levels of Th1/Th2/Treg/Th17-related cytokines. Collectively, these findings indicate that JPBY ameliorates spleen-deficiency-type AR while simultaneously remodeling the gut microbiota and SCFA metabolism, improving intestinal and nasal mucosal barrier function, and attenuating TLR4/NF-κB-associated inflammation. The coordinated alterations observed across the gut and nasal compartments suggest that gut–nose axis-related mechanisms may be involved in the effects of JPBY, although a direct causal relationship remains to be established.

## 1 Introduction

Allergic rhinitis (AR) is a chronic, non-infectious inflammatory disease of the nasal mucosa mediated primarily by immunoglobulin E (IgE) and is classified as a type I hypersensitivity disorder. With a global prevalence ranging from 5% to 50%, AR imposes a substantial personal and socioeconomic burden, which is expected to increase further as its prevalence continues to rise (1). Consequently, AR has become an increasingly important global public health concern. The main clinical manifestations of AR include sneezing, rhinorrhea, nasal itching, and nasal congestion. When inadequately controlled, these symptoms may significantly impair patients’ quality of life and can contribute to psychological distress, sleep disturbances, and reduced work productivity (2). The pathogenesis of AR is complex and multifactorial. Although the IgE-mediated inflammatory response remains the predominant paradigm, accumulating evidence indicates that AR also involves non-IgE-mediated inflammatory processes, including the infiltration and activation of various immune cells, disruption of the epithelial barrier, and alterations in the nasal microbiota (1). An imbalance in cytokine production by Th1 and Th2 cells has traditionally been regarded as a key mechanism underlying AR, while dysregulation of the Treg/Th17 axis has emerged as an important complementary mechanism (3). In addition, the integrity of the nasal mucosal barrier plays a critical role in preventing the penetration of allergens and pathogenic microorganisms while maintaining local immune homeostasis (4). Impairment of the epithelial barrier is characterized, among other changes, by reduced expression of tight junction (TJ) proteins such as occludin and zonula occludens-1 (ZO-1), which may facilitate persistent inflammation in allergen-exposed tissues (5). Although substantial advances have been made in elucidating the pathophysiology of AR, its complex and interconnected pathogenic mechanisms remain incompletely understood.

It is noteworthy that the relationship between the gut microbiota and respiratory diseases has increasingly attracted attention, and the emerging concept of the “gut–respiratory axis” has provided a novel perspective for understanding the pathogenesis of AR (6, 7). From the perspective of traditional Chinese medicine (TCM), spleen deficiency is considered one of the fundamental pathogenic mechanisms underlying AR. In addition to typical nasal manifestations, patients with spleen-deficiency-type AR often present with systemic symptoms, including fatigue, reduced appetite, abdominal distension, and loose stools. This subtype is also characterized by a prolonged disease course and a high tendency for recurrence (8). According to TCM theory, the spleen is responsible for the transformation and transportation of nutrients and body fluids. Spleen deficiency may result in impaired distribution of the essence derived from food and fluids, as well as insufficient nourishment of the lung-wei (defensive qi), which shares certain conceptual similarities with intestinal barrier disruption and microbiota dysregulation described in modern medicine (9). Based on the TCM theories that “the lung and large intestine are internally and externally related” and that “the spleen governs transformation and transportation,” together with advances in microbiome research, the “gut–nose axis” has been proposed as a potential regulatory network involved in the initiation and progression of AR through multiple pathways, including immune modulation, microbial metabolism, and neuroendocrine regulation (7, 10). Accumulating evidence indicates that both AR patients and corresponding animal models exhibit alterations in the composition and functional capacity of the gut microbiota (11, 12). Gut microbial dysbiosis can compromise intestinal barrier integrity and immune tolerance, promote Th2-predominant immune responses, and disturb the Th1/Th2 and Treg/Th17 immune balance, thereby aggravating IgE-mediated type 2 inflammatory responses (13). Microbial metabolites represent critical mediators in the communication between the gut microbiome and the host immune system. Among these metabolites, short-chain fatty acids (SCFAs) are essential for maintaining intestinal barrier homeostasis, facilitating Treg cell differentiation, and limiting excessive inflammatory activation. In patients with AR, SCFA-producing bacterial taxa are decreased, whereas potentially inflammation-associated microbial populations are relatively enriched (11). Such alterations may result in reduced SCFA production, thereby contributing to immune dysregulation and exacerbating inflammation of the nasal mucosa. Therefore, it has been proposed that spleen-deficiency-type AR may involve a continuous pathological cascade characterized by “spleen dysfunction–gut microbiota dysbiosis–metabolic disturbance–immune imbalance–nasal mucosal inflammation.” In recent years, therapeutic strategies targeting the gut microbiota, including probiotics, TCM-based interventions, and fecal microbiota transplantation, have shown potential in alleviating AR symptoms (7, 14, 15). Thus, investigating the pathogenesis of spleen-deficiency-type AR through the framework of the “gut–nose axis” may provide new theoretical insights and identify potential therapeutic targets for the precise prevention and management of AR.

The formula of JianpiBiyan (JPBY) is an empirical formula used in TCM to treat AR; most of its ingredients possess properties that are common to both food and medicine. The herbal composition of JPBY is a modified version of the “Jiegen Yuanshen Decoction,” a formula that has been used in TCM to treat nasal disorders for over 200 years. Modern research has further confirmed that this classic formula has a clear therapeutic effect on AR symptoms, with both relevant clinical trials and animal studies verifying its efficacy (16–18). JPBY is composed of 10 herbs, *Poria cocos* (Schw.) Wolf and *Atractylodes macrocephala* Koidz. dispel water-dampness and strengthen the spleen and stomach. *Angelica dahurica* (Fisch.ex Hoffm.) Benth.et Hook.f. and *Magnolia biondii* Pamp. promote nasal ventilation. *Pinellia ternata* (Thunb.) Breit. and *Citrus reticulata Blanco* promote digestion in the stomach and intestines and dry dampness and resolve phlegm. *Platycodon grandiflorum* (Jacq.) A.DC. and *Prunus armeniaca* L.var.ansu Maxim. can moisten the lungs, dissolve phlegm and promote bowel movements. *Zingiber officinale* Rosc. disperses cold and relieves the symptoms, and *Glycyrrhiza uralensis* Fisch. replenishes spleen and stomach and balances the efficacy. The formula is based on strengthening the spleen, tonifying qi, drying dampness, and resolving phlegm, while also promoting lung function and unblocking the orifices. It aligns closely with the pathogenic mechanisms of spleen-deficiency-type AR, characterized by “spleen deficiency leading to impaired transport and transformation, internal generation of phlegm-dampness, and instability of the lung-wei defense.” It is worth noting that many of the herbs in JPBY are classified as “medicinal foods” or have a history of long-term dietary use. Their advantages lie not only in their ability to regulate the body holistically and their potential for effective long-term intervention but also in their possible role in improving the gut microbiome (19). Modern research indicates that polysaccharides found in traditional TCM that strengthen the spleen—such as Baizhu-can be utilized by the gut microbiota, promoting the accumulation of beneficial microbial metabolites, enhancing the integrity of the intestinal epithelial barrier, and regulating mucosal immunity (20); dietary polysaccharides found in citrus-based medicinal materials, such as Chenpi, also have the potential to regulate the composition of the gut microbiota, maintain the intestinal mucosal barrier, and suppress inflammatory responses (21). These effects may help restore the impaired intestinal microbiota and immune homeostasis associated with spleen deficiency. Existing research further indicates that TCM treatment for AR exerts a multi-target effect by regulating the gut microbiota, improving intestinal barrier function, and correcting Th1/Th2 and Treg-related immune imbalances (7, 22). Therefore, JPBY may intervene in spleen-deficiency-type AR through a synergistic pathway of “strengthening the spleen—regulating the gut microbiota—improving microbial metabolism and the intestinal barrier—modulating immunity,” which also highlights the potential advantages of TCM derived from food-medicine homology in the long-term management of chronic, recurrent diseases. However, whether JPBY exerts its effects through the aforementioned “gut-nose axis” mechanism, as well as its key microbial communities and metabolites, remains to be further verified.

Based on the above considerations, we hypothesized that the therapeutic effects of JPBY on spleen-deficiency-type AR may be associated with the coordinated modulation of gastrointestinal function, the gut microbiota and its metabolites, mucosal barrier integrity, and immune-inflammatory signaling, potentially involving the gut–nose axis. To test this hypothesis, we established a rat model of spleen-deficiency AR and systematically evaluated the effects of JPBY at multiple levels, including gastrointestinal function, intestinal and nasal mucosal barrier integrity, gut microbiota and its metabolites, and immune-inflammatory responses. Multi-omics approaches, including gut microbiome profiling, SCFA metabolomics, and transcriptomic analysis, were integrated to explore the underlying mechanisms. Furthermore, UHPLC-MS/MS, network pharmacology, molecular docking, and molecular biology experiments were combined to identify and validate the absorbed constituents of JPBY, potential therapeutic targets, and key molecular pathways. By integrating these multidimensional lines of evidence, this study aimed to elucidate the potential biological mechanisms underlying the protective effects of JPBY against spleen-deficiency AR and to further investigate the potential links among gut microbiota and microbial metabolites, mucosal barrier integrity, and nasal immune-inflammatory responses, thereby providing experimental evidence for understanding the biological association between spleen deficiency and AR from the perspective of the gut–nose axis.

## 2 Materials and methods

### 2.1 Materials and chemicals

All ingredients of JPBY and the Da Huang (*Rheum palmatum L.*) required for inducing spleen deficiency in rats were purchased from China Resources Sanjiu Modern Chinese Medicine Pharmaceutical Co., Ltd. The corresponding lot numbers are recorded to ensure traceability, and the quality of each herbal medicine is controlled in accordance with the relevant national pharmaceutical standards (Jiegeng: 2212007C, Kuxingren: 2302001S, Fuling: 2302002S, Baizhu: 2211051C, Chenpi: 2208015S, Banxia: 2306002C, Shengjiang: 2306004C, Xinyi: 2305003W, Baizhi: 2307003C, Gancao: 2204019C, Dahuang: 2210001C). JPBY was prepared according to the prescription composition and daily dosage listed in **Table 1**, which represents the total daily dose of JPBY for an adult.

**Table 1:** Composition of Jianpi Biyan formula.

No.	Chinese name	Family of the plant	Herbal name	Part used	Dose(g)
1	Jiegeng	Eustomaaceae	Platycodon grandiflorum (Jacq.) A.DC.	Radix	9
2	Kuxingren	Rosaceae	Prunus armeniaca L.var.ansu Maxim.	Semen	9
3	Fuling	Polyporaceae	Poria cocos (Schw.) Wolf	nucleus (of fungus)	9
4	Baizhu	Compositae	Atractylodes macrocephala Koidz.	Rhizoma	9
5	Chenpi	Rutaceae	Citrus reticulata Blanco	Pericarpium	9
6	Banxia	Araceae	Pinellia ternata (Thunb.) Breit.	Rhizoma	9
7	Shengjiang	Gingeraceae	Zingiber officinale Rosc.	Rhizoma	9
8	Xinyi	Magnoliaceae	Magnolia biondii Pamp.	Buds	9
9	Baizhi	Apiaceae	Angelica dahurica (Fisch.ex Hoffm.) Benth.et Hook.f.	Radix	9
10	Gancao	Fabaceae	Glycyrrhiza uralensis Fisch.	Radix or Rhizoma	6

Ovalbumin (OVA, S12015) was purchased from Shanghai yuanye Bio-Technology Co., Ltd. (Shanghai, China). Al(OH)_3_ (239186) was purchased from Sigma-Aldrich LLC. (Shanghai, China). Loratadine tablets (12202046077) were purchased from Hainan Haishen Tongzhou Pharmaceutical Co., Ltd. (Hainan, China). Occludin antibody (ab216327) was purchased from abcam Trading Co., Ltd. (Shanghai, China). ZO-1 antibody (PTM-5165) and TLR4 antibody (PTM-5192) were purchased from Jingjie Bioscience Co., Ltd. (Guangzhou, China). MyD88 antibody(67969), NF-κB-p65 antibody(10745), p-NF-κB-p65 antibody(82335), β-actin antibody (66009)were purchased from Proteintech Group, Inc (Wuhan, China). ELISA Kits for OVA-sIgE, IFN-γ, TNF-α, IL-6, IL-1β, IL-10, and IL-17 were purchased from Huangshi Yankebio Co., Ltd. (Huangshi, China). ELISA kits for pancreatic lipase, trypsin, gastrin, and motilin were purchased from Shanghai Enzyme-Linked Biotechnology Co., Ltd. (Shanghai, China).

### 2.2 Animals

Male Sprague-Dawley rats were acquired from Liaoning Changsheng Biotechnology Co., Ltd. (Liaoning, China). Those that passed quarantine and exhibited no abnormal behavior were included in the experiment. Animals that died within 24 hours after model induction or developed severe complications unrelated to the experiment were excluded from the study, and the reasons for exclusion were recorded in detail. The Animal Ethics Committee of Changchun University of Chinese Medicine approved all animal experiments (No. 2024721). Adaptive feeding was performed for 7 days under standard laboratory conditions with controlled environmental conditions: constant temperature of 22 ± 2°C, relative humidity of 55 ± 5%, ad libitum diet, and 12 h light/dark cycle. Rats were assigned to groups in a completely randomized manner using a random number table, resulting in a total of 7 groups (6 rats per group): rats from the JPBY drug-containing serum group (JPBYDS) and the control serum group were used to prepare JPBY drug-containing serum; the control group (CON), model group (MOD), the JPBY low-dose group (JPBY-L), the JPBY high-dose group (JPBY-H), and loratadine group (LOR) were used to investigate the therapeutic effects of JPBY on rats with spleen-deficiency-associated AR. This study employed an analyst-blind design.

### 2.3 Preparation of JPBY drug-containing serum

Rats in the JPBYDS group were administered JPBY by gavage at a dose of 9.06 g/kg, whereas rats in the control serum group received an equal volume of 0.9% sodium chloride solution by gavage. All rats were gavaged twice daily for 7 consecutive days. One hour after the final administration, the rats were anesthetized with isoflurane, and blood samples were collected. The blood samples were allowed to stand at room temperature for 1 h, followed by centrifugation at 3,500 rpm for 10 min at 4 °C to separate the serum. The separated serum samples were stored at −80 °C until further analysis.

### 2.4 Establishment of an AR rat model with spleen deficiency syndrome

Rats in the MOD, JPBY-L, JPBY-H, and LOR groups were sensitized with OVA to establish an AR model, as previously described (23). Briefly, a suspension containing 0.3 mg of OVA and 30 mg of Al(OH)_3_ in 1 mL of saline was prepared and administered by intraperitoneal injection once every other day for 14 days. After the final intraperitoneal injection, local nasal challenge was initiated. A 10% OVA saline suspension was administered into both nasal cavities once daily for 7 consecutive days (20 μL per nostril).

During the AR modeling period, the “bitter-cold purgation method” combined with the “irregular eating pattern method” was used to establish the spleen deficiency syndrome model (24). Rats were administered a 15% Da Huang powder suspension by gavage at a dose of 5 mL once daily for 21 consecutive days (25). During the modeling period, the rats were provided with approximately 50% of their normal daily food intake, while water was provided ad libitum.

### 2.5 Assessment of rat symptoms and model validation

Nasal symptoms were assessed at two time points: after completion of model induction and after completion of the final drug treatment. Following the establishment of the AR model, nasal symptoms were evaluated to determine the success of model induction. Rats were observed for 10 min, during which sneezing, nose-rubbing, and nasal discharge were recorded. Sneezing and nose-rubbing behaviors were scored as follows: 0 points, no occurrence; 1 point, 1–3 occurrences per min; 2 points, 4–6 occurrences per min; and 3 points, >6 occurrences per min. Nasal discharge was scored as follows: 0 points, no discharge; 1 point, discharge from one nostril; 2 points, discharge from both nostrils; and 3 points, excessive discharge with spillage. The three symptom scores were summed to obtain the total nasal symptom score for each rat. A total score ≥5 was considered indicative of successful establishment of the AR model (23). After completion of the final drug treatment, the nasal symptoms of the rats were assessed again using the same scoring criteria to evaluate the effects of the respective treatments on AR-related symptoms.

The spleen deficiency syndrome model was evaluated according to previously reported criteria (26). The scoring criteria were as follows: (1) huddling, reduced activity, and clustering, 1 point; (2) arched back, narrowed eyes, drowsiness, lethargy, and dry or brittle fur, 1 point; (3) reduced food and water intake, 1 point; (4) slow weight gain, weight loss, or emaciation compared with the previous observation period, 2 points; (5) poor perianal hygiene and soft or loose stools, 2 points; (6) decreased rectal temperature, 1 point; and (7) reduced swimming endurance, 2 points. In addition, the spleen deficiency model was further evaluated in terms of gastrointestinal digestive function by measuring the levels of gastrointestinal hormones (gastrin, motilin) and digestive enzymes (pancreatic lipase, trypsin) in rats (27).

### 2.6 Drug intervention procedures

After completion of the 7-day local nasal challenge, nasal symptoms were assessed to confirm successful establishment of the spleen deficiency syndrome-type AR model. Rats in the JPBY-L, JPBY-H, and LOR groups then received drug treatment for 2 weeks. The clinically equivalent dose for rats was calculated based on body surface area conversion between humans and rats, using an equivalent dose conversion factor of 6.25 for rats (28). Rats in the JPBY-L group received JPBY at a dose of 9.06 g/kg, corresponding to the clinically equivalent dose, whereas rats in the JPBY-H group received JPBY at a dose of 27.18 g/kg, corresponding to three times the clinically equivalent dose. JPBY was administered by gavage twice daily. Rats in the LOR group received loratadine at a dose of 1 mg/kg by gavage once daily. Rats in the CON and MOD groups received an equal volume of 0.9% sodium chloride solution by gavage.

### 2.7 Identification of the major components of JPBY absorbed into the bloodstream

To identify the major components of JPBY that are absorbed into the bloodstream, UHPLC-MS/MS was performed to analyze the prepared JPBY herbal medicine, JPBY-containing serum, and control serum. Four samples were analyzed for each sample type. Briefly, 100 mg of the prepared JPBY herbal medicine was extracted with a methanol-containing extraction solution. After vortex mixing, ultrasonication in an ice-water bath, and centrifugation, the resulting supernatant was filtered and pooled to prepare a quality control (QC) sample for subsequent analysis. For serum sample preparation, 400 μL of serum collected from rats in the JPBYDS or control serum group was mixed with hydrochloric acid. After repeated vortex mixing and incubation, acetonitrile was added for protein precipitation. The samples were vortex-mixed and centrifuged, and the resulting supernatants were collected and evaporated to dryness under a stream of nitrogen. The residues were reconstituted in methanol, vortex-mixed, and centrifuged. The resulting supernatants were collected for UHPLC-MS/MS analysis.

Chromatographic separation was performed using an ACQUITY UPLC BEH C18 column (1.7 μm × 2.1 mm × 100 mm; Waters Corporation, Milford, MA, USA; P/N 186002352) coupled to a Vanquish UHPLC system (Thermo Fisher Scientific, Waltham, MA, USA). The flow rate was set at 0.5 mL/min, and the injection volume was 5 μL. The mobile phase consisted of 0.1% formic acid in water (A) and 0.1% formic acid in acetonitrile (B). The linear gradient elution program was as follows: 0–11 min, 85–25% A; 11–12 min, 25–2% A; 12–14 min, 2% A; 14–14.1 min, 2–85% A; and 14.1–16 min, 85% A. Mass spectrometric analysis was performed using an Orbitrap Exploris 120 mass spectrometer (Thermo Fisher Scientific, Waltham, MA, USA) controlled by Xcalibur software. MS and MS/MS data were acquired in information-dependent acquisition (IDA) mode. The mass range was set to m/z 100–1500. In each acquisition cycle, the four most intense precursor ions were selected for subsequent MS/MS acquisition. The MS parameters were set as follows: sheath gas flow rate, 35 Arb; auxiliary gas flow rate, 15 Arb; ion transfer tube temperature, 350 °C; vaporizer temperature, 350 °C; full MS resolution, 60,000; MS/MS resolution, 15,000; normalized collision energy (NCE), 16/32/48; and spray voltage, 5.5 kV in positive ion mode and −4.0 kV in negative ion mode. The major components of JPBY absorbed into the bloodstream were identified by comparing the chromatographic and mass spectrometric profiles of the prepared JPBY herbal medicine, JPBY-containing serum, and control serum. Components detected in both the JPBY preparation and JPBY-containing serum but absent from the control serum were considered candidate absorbed components of JPBY. These candidate absorbed components were subsequently used as the basis for network pharmacology analysis, molecular docking, and further mechanistic investigations.

### 2.8 Gut microbiota analysis

After treatment, cecal contents were collected from the CON, MOD, and JPBY-H groups. Based on predefined sequencing quality criteria, 5 samples per group were included in the final analysis. Genomic DNA was extracted, and its concentration, purity, and integrity were assessed using a NanoDrop spectrophotometer (Thermo Fisher Scientific, A30221) and agarose gel electrophoresis. The V3–V4 region of the bacterial 16S rRNA gene was amplified using primers 341F (5′-CCTACGGGNGGCWGCAG-3′) and 805R (5′-GACTACHVGGGTATCTAATCC-3′). PCR products were purified with AMPure XT beads (Beckman Coulter Genomics, Danvers, MA, USA), quantified using a Qubit fluorometer (Invitrogen, USA), and evaluated using an Agilent 2100 Bioanalyzer and KAPA Library Quantification Kit (Kapa Biosystems, Woburn, MA, USA). Qualified libraries were subjected to paired-end sequencing on the Illumina NovaSeq PE250 platform.

Raw sequences were merged, quality-filtered, and screened for chimeras, followed by DADA2 denoising to generate amplicon sequence variants (ASVs) (29, 30). Taxonomic classification was performed against the SILVA (Release 138) and NT-16S databases using a confidence threshold of >0.7. The resulting ASV table and taxonomic annotations were used for downstream diversity and differential abundance analyses. Alpha diversity was assessed using the Chao1 index, whereas beta diversity was evaluated using Bray–Curtis distances and visualized by principal coordinates analysis (PCoA). Differential taxa among groups were identified using the Kruskal–Wallis test, and LEfSe analysis was subsequently performed using the Kruskal–Wallis and Wilcoxon rank-sum tests, with an LDA score>2 and P<0.05 considered indicative of discriminatory taxa.

### 2.9 Targeted metabolomic analysis of SCFAs

Cecal contents were collected from rats in the MOD and JPBY-H groups for targeted metabolomic analysis of SCFAs, with 5 samples analyzed per group. Metabolite extraction was performed as follows: 100 mg of each sample was resuspended in 20% phosphoric acid, mixed with isopropyl ether, and centrifuged. The resulting supernatant was collected for subsequent GC-MS/MS analysis. Chromatographic separation was performed using an Agilent DB-FFAP capillary column (30 m × 0.25 mm × 0.25 μm; Agilent Technologies, Santa Clara, CA, USA; P/N 122-3232). The initial column temperature was set at 90 °C and increased to 160 °C at a rate of 10 °C/min, followed by an increase to 240 °C at 40 °C/min, where it was maintained for 5 min. Helium was used as the carrier gas at a flow rate of 1.0 mL/min. QC samples were analyzed to evaluate the stability and repeatability of the analytical system. Mass spectrometric analysis was performed using a 5977B GC/MSD system (Agilent Technologies, Santa Clara, CA, USA). The MS conditions were as follows: inlet temperature, 250 °C; ion source temperature, 230 °C; transfer line temperature, 250 °C; and quadrupole temperature, 150 °C. Electron impact (EI) ionization was performed at an energy of 70 eV. Mass spectrometric data were acquired in selected ion monitoring (SIM) mode.

### 2.10 Network pharmacology analysis and molecular docking

Potential target genes of the major components of JPBY absorbed into the bloodstream and SCFAs were predicted using the SEA Search Server database (https://sea.bkslab.org/). AR-related target genes were identified using the DisGeNET database (http://www.disgenet.org/) and the GeneCards database (http://www.genecards.org/). The gutMGene database (https://bio-computing.hrbmu.edu.cn/gutmgene/#/home) was used to obtain target genes associated with the differential gut microbiota identified in this study. The target genes associated with the absorbed components of JPBY, differential gut microbiota, SCFAs, and AR were integrated and compared, and overlapping targets were identified. These overlapping targets were considered potential key targets through which JPBY may regulate the gut–nose axis in the treatment of AR. The overlapping targets were imported into the STRING database (https://string-db.org/) to construct a protein–protein interaction (PPI) network. The PPI network was subsequently visualized and analyzed using Cytoscape 3.8.2. Core target genes were identified based on network topology characteristics. Gene Ontology (GO) and Kyoto Encyclopedia of Genes and Genomes (KEGG) pathway enrichment analyses were then performed to further characterize the biological functions and signaling pathways associated with the core target genes. Molecular docking was subsequently performed to investigate the potential interactions between the identified key components and core target proteins (31). Xanthotoxol and arctigenin were selected as representative ligand molecules, whereas TLR4, MyD88, NF-κB, and IL-6 were selected as receptor proteins. The three-dimensional structures of the receptor proteins were obtained from the Protein Data Bank (PDB) database (https://www.rcsb.org/), and the molecular structures of the ligand compounds were obtained from the PubChem database (https://pubchem.ncbi.nlm.nih.gov/). The receptor and ligand structures were subsequently prepared using AutoDock Tools and subjected to molecular docking analysis to evaluate their potential binding affinities and interaction modes.

### 2.11 Transcriptome sequencing

Nasal mucosal tissue samples were randomly selected from three rats in each of the CON, MOD, and JPBY-H groups for transcriptome sequencing. The experimental workflow was as follows. First, total RNA was extracted from the nasal mucosal tissues and subjected to quality assessment. Messenger RNA (mRNA) was purified from the total RNA using oligo(dT) magnetic beads and subsequently fragmented. The fragmented mRNA was used as a template for reverse transcription to synthesize complementary DNA (cDNA), followed by PCR amplification. The amplified products were purified, and the resulting sequencing libraries were subjected to quality assessment prior to sequencing. Following sequencing, adapter sequences were removed, and low-quality reads and reads containing ambiguous bases (N) were filtered out to obtain high-quality clean reads. Sequencing quality was assessed based on Q20, Q30, N content, and the proportion of clean reads. Sequencing depth was evaluated to ensure sufficient coverage for subsequent transcript expression analysis. Differential gene expression analysis was performed using the DESeq2 package. Genes with an adjusted p value <0.05 and an absolute log2 fold change (|log2FC|) >1 were considered differentially expressed genes (DEGs). DEGs were identified through pairwise comparisons between the CON and MOD groups and between the MOD and JPBY-H groups. Venn diagrams were generated to identify overlapping DEGs between the two pairwise comparisons. Among the overlapping DEGs, genes exhibiting opposite expression trends in the two comparisons were identified as candidate genes associated with the reversal of disease-related transcriptional changes by JPBY-H. Finally, functional annotation and enrichment analyses were performed for the overlapping DEGs exhibiting opposite expression trends.

### 2.12 ELISA

Small intestinal tissues were collected from rats in each group, homogenized, and centrifuged to obtain the supernatants. The concentrations of pancreatic lipase and trypsin in the small intestinal tissue supernatants were determined using ELISA kits according to the manufacturers’ instructions. Serum levels of OVA-sIgE, IFN-γ, TNF-α, IL-6, IL-1β, IL-10, IL-17, gastrin, and motilin were also measured using ELISA kits according to the manufacturers’ instructions. These measurements were performed to assess alterations in gastrointestinal digestive function and immune-inflammatory status in rats with spleen deficiency syndrome and to evaluate the therapeutic effects of JPBY.

### 2.13 H&E, immunohistochemical, and immunofluorescence staining

Nasal mucosal and colonic tissues were collected from rats, fixed in 4% paraformaldehyde, paraffin-embedded, and sectioned at a thickness of 4 μm. Nasal mucosal and colonic sections were stained with hematoxylin and eosin (H&E) to evaluate the histopathological morphology of the tissues. Immunohistochemical staining was performed on nasal mucosal and colonic sections to evaluate the expression of ZO-1 and occludin. Immunofluorescence staining was performed on nasal mucosal sections to evaluate the expression of TLR4 and MyD88. Images were captured using a light microscope at 200× magnification. Image-Pro Plus 6.0 software was used to quantify the average optical density (OD) or relative fluorescence intensity, as appropriate.

### 2.14 Western blot analysis

The protein expression levels of TLR4, MyD88, NF-κB p65, and phosphorylated NF-κB p65 (p-NF-κB p65) in the nasal mucosal and colonic tissues of rats in each group were analyzed by Western blotting. For protein extraction, 50 mg of nasal mucosal tissue or 30 mg of colonic tissue from each rat was homogenized in lysis buffer, and total protein was extracted. Protein concentrations were determined using an appropriate protein assay. Equal amounts of protein (20 μg per sample) were separated by 10% SDS-PAGE and transferred onto polyvinylidene difluoride (PVDF) membranes. The membranes were blocked with 5% skim milk for 1 h at room temperature and then incubated overnight at 4 °C with the following primary antibodies: anti-TLR4 (1:1,000), anti-MyD88 (1:3,000), anti-NF-κB p65 (1:4,000), and anti-p-NF-κB p65 (1:5,000). After washing three times with TBST, the membranes were incubated with the corresponding secondary antibodies for 1 h at room temperature. The membranes were subsequently washed three times with TBST, and the protein bands were visualized and imaged.

### 2.15 Statistical analysis

Statistical analysis was performed using GraphPad Prism (8.0) and R (4.5.0) software. Data from animal experiments are expressed as mean ± standard deviation (SD). Prior to statistical analysis, the data were tested for normality of distribution and homogeneity of variances. If the data were normally distributed and had homogeneous variances, one-way analysis of variance (ANOVA) was performed, followed by Tukey’s multiple comparison test; if the data did not meet these assumptions, the Kruskal–Wallis test was performed, followed by Dunn’s multiple comparison test. All statistical tests were two-sided, and *P<0.05* was considered statistically significant. For the analysis of transcriptomic and 16S rRNA data, the Benjamini–Hochberg correction method was used for multiple comparison correction to control the false discovery rate (FDR). In the analysis of differential expression or differential abundance, an adjusted *P-value (FDR)<0.05* was considered statistically significant.

## 3 Results

### 3.1 JPBY ameliorates the clinical and spleen-deficiency-related phenotypes in AR rats

To evaluate the therapeutic effects of JPBY on spleen-deficiency-type AR, a rat model integrating both disease and TCM syndrome characteristics was established (**Figure 1A**). Following model induction, the rats were treated with different doses of JPBY or LOR for 2 consecutive weeks. Compared with the CON group, body weight was significantly reduced in the modeled rats (*P<0.01*). However, treatment with high-dose JPBY significantly increased body weight compared with the MOD group (*P<0.01*; **Figure 1B**). The nasal symptom scores and spleen-deficiency symptom scores before and after treatment are presented in **Figures 1C, D**, respectively. Following treatment, both nasal and spleen-deficiency symptoms were alleviated to varying degrees. Notably, compared with LOR treatment, JPBY produced a more pronounced improvement in symptoms, with the therapeutic benefit increasing in a dose-dependent manner. In addition, serum OVA-specific IgE (OVA-sIgE) levels were significantly lower in the JPBY-H group than in the MOD group (*P<0.01*; **Figure 1E**). To evaluate the effect of JPBY on improving gastrointestinal digestive function in rats with spleen-deficiency-type AR, changes in gastrointestinal hormone and digestive enzyme-related indicators—including gastrin, motilin, pancreatic lipase, and trypsin levels—were further assessed. The results showed that, compared with the CON group, serum gastrin and motilin levels in rats in the MOD group were both significantly lower (*P<0.01*; **Figures 1F, G**), suggesting that gastrointestinal hormone secretion was abnormal after model induction, and that gastrointestinal motility and digestive function were significantly affected. In addition, pancreatic lipase and trypsin levels in the small intestine tissue of rats in the MOD group were both significantly reduced (*P<0.01*; **Figures 1H, I**), further indicating that AR rats with spleen deficiency exhibit marked digestive enzyme secretion disorders and impaired digestive function. Following JPBY intervention, the aforementioned abnormalities were improved to varying degrees. Compared with the MOD group, JPBY treatment significantly increased serum gastrin and motilin levels in rats and elevated pancreatic lipase and trypsin levels in small intestinal tissue, with the JPBY-H group showing the most significant improvement (*P<0.01*). These results indicate that JPBY can effectively improve abnormal gastrointestinal motility and digestive enzyme secretion in spleen-deficiency-type AR rats, with high-dose JPBY demonstrating a more pronounced therapeutic effect.

**Figure 1:**
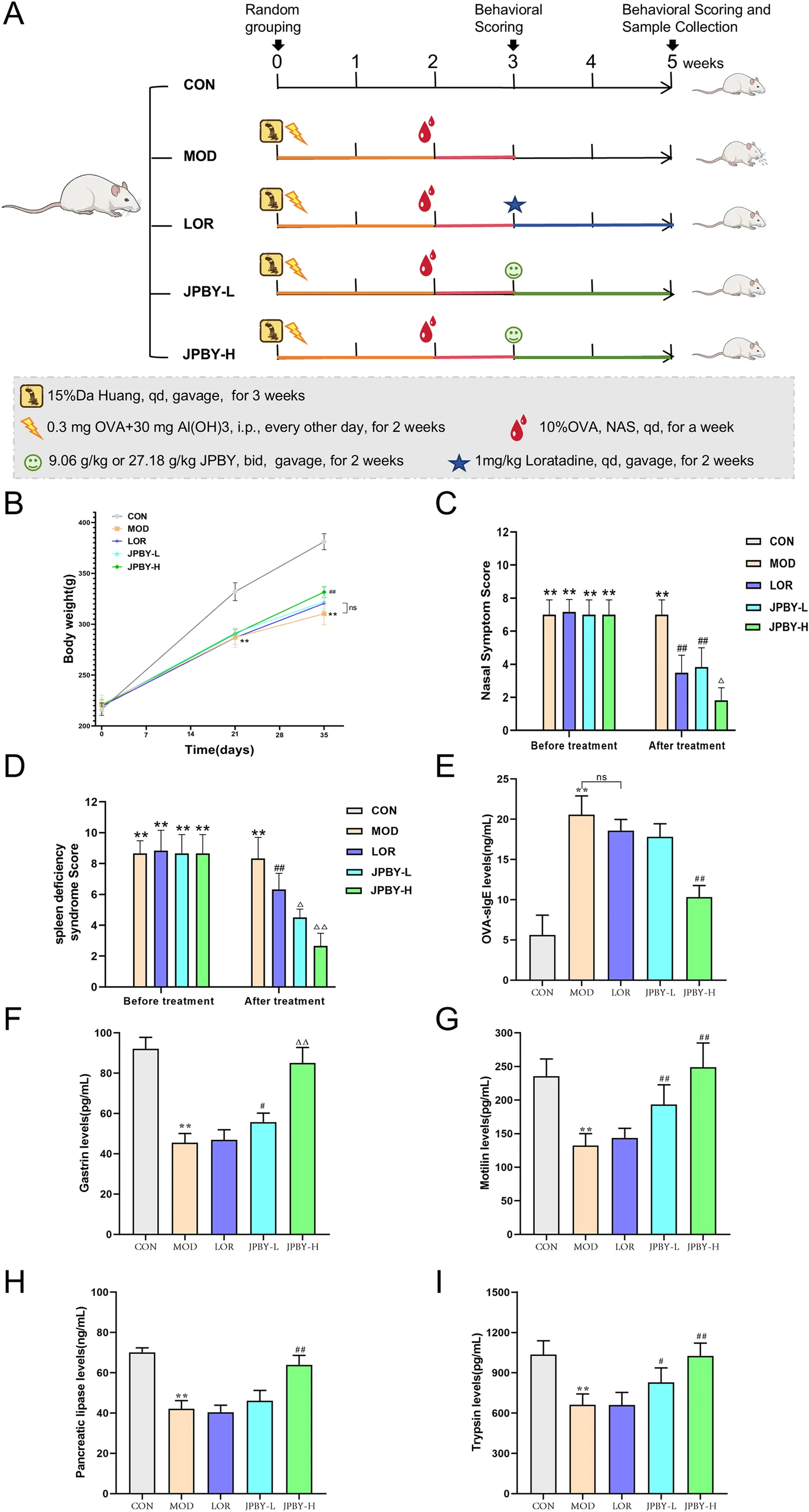
JPBY ameliorates the clinical and spleen-deficiency-related phenotypes in AR rats. **(A)** Flowchart for animal experiments. **(B)** Boby weight of rats in each group. **(C)** Nasal symptom scores of rats in each group. **(D)** Spleen deficiency symptom scores of rats in each group. **(E)** Serum OVA-sIgE levels in rats of each group. **(F, G)** Serum gastrin and motilin levels in rats of each group. **(H, I)** Levels of pancreatic lipase and trypsin in small intestine tissues from rats in each group. Values are presented as the mean ± SD. **p<0.01, *p<0.05, vs. the CON group. *^##^p<0.01*, *^#^p<0.05* vs. the MOD group. *^ΔΔ^p<0.01*, *^Δ^p<0.05* vs. the LOR group.

### 3.2 JPBY can alleviate pathological damage in rats with spleen-deficiency-type AR and restore the integrity of the intestinal and nasal mucosal barriers

H&E staining revealed marked pathological alterations in the nasal mucosa of rats in the MOD group, including turbinate swelling, pronounced disruption of the nasal mucosal architecture, disorganized ciliary arrangement, and extensive inflammatory cell infiltration (**Figure 2A**). Compared with the MOD group, both JPBY-L and JPBY-H treatment alleviated these pathological changes to varying degrees. Notably, the JPBY-H group exhibited a more pronounced reduction in inflammatory cell infiltration and a relatively well-preserved and orderly ciliary arrangement. In the colon, rats in the MOD group showed extensive inflammatory cell infiltration accompanied by disruption and loss of the normal intestinal crypt architecture. Following JPBY treatment, these pathological abnormalities were alleviated, indicating a reparative effect on colonic tissue injury. Immunohistochemical analysis further showed that the expression levels of the tight junction proteins ZO-1 and occludin in both the nasal mucosa and colonic tissues were significantly lower in the MOD group than in the CON group (*P<0.01*). Compared with the MOD group, JPBY treatment significantly increased the expression of ZO-1 and occludin in the nasal mucosa (*P<0.01*). In colonic tissues, JPBY increased the expression of both proteins in a dose-dependent manner, with statistically significant differences observed at *P<0.05* or *P<0.01* (**Figures 2B–F**). These findings suggest that JPBY helps preserve the integrity of both the nasal and intestinal mucosal barriers and alleviates associated pathological tissue damage.

**Figure 2:**
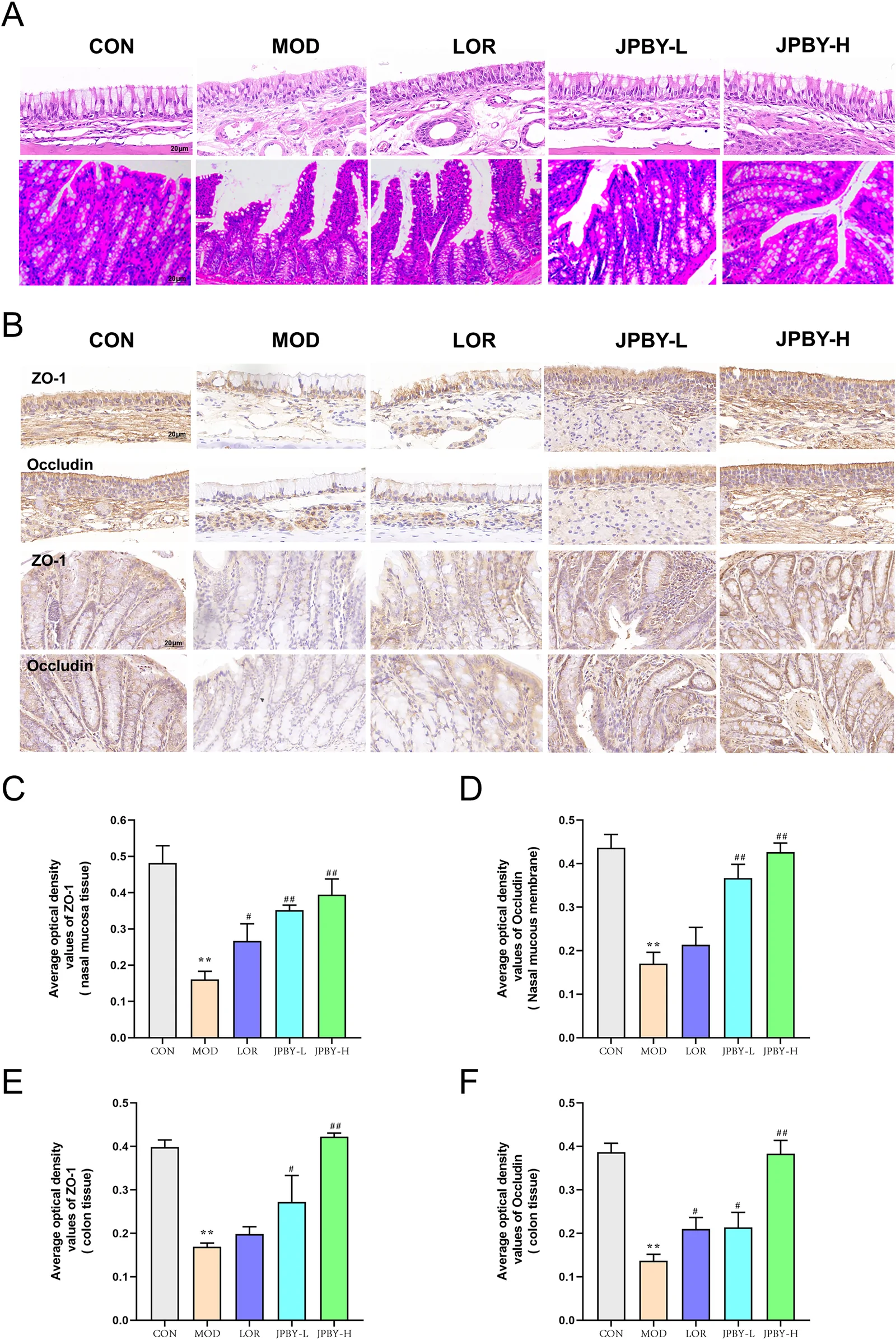
JPBY can alleviate pathological damage in rats with spleen-deficiency-type AR and restore the integrity of the intestinal and nasal mucosal barriers. **(A)** H&E staining of nasal mucosa and colon in rats from each group (scale bar: 20 μm). **(B)** Immunohistochemical staining images of ZO-1 and Occludin in the nasal mucosa and colon of rats from each group (scale bar: 20 μm). **(C–F)** Statistical results for average optical density values. Values are presented as the mean ± SD. **p<0.01, *p<0.05, vs. the CON group. *^##^p<0.01*, *^#^p<0.05* vs. the MOD group.

### 3.3 JPBY modulates gut microbiota composition and SCFA profiles in AR rats with spleen deficiency

To investigate the regulatory effects of JPBY on the gut microbiota, cecal contents from rats in the JPBY-H, CON, and MOD groups were subjected to 16S rRNA sequencing. Analysis of microbial diversity showed that the MOD group had a significantly reduced Chao1 index, accompanied by a downward trend in overall microbial diversity, whereas JPBY treatment partially restored microbial diversity (**Figures 3A, B**). At both the phylum and species levels, the composition and relative abundance of dominant microbial taxa in the JPBY-H group differed markedly from those in the MOD group (**Figures 3C, D**). LEfSe analysis further revealed that, compared with the MOD group, JPBY-H treatment significantly increased the relative abundances of Ligilactobacillus, Lachnospiraceae, and Bacteroidales, while reducing those of Christensenellaceae and Escherichia (**Figure 3E**). In addition, targeted metabolomic analysis of SCFAs demonstrated that the levels of acetic acid, propionic acid, butyric acid, and isobutyric acid were significantly higher in the JPBY-H group than in the MOD group (**Figures 3F–I**; *P<0.05* or *P<0.01*). Collectively, these findings suggest that JPBY treatment modulated the gut microbial community and was associated with increased levels of several SCFAs in AR rats.

**Figure 3:**
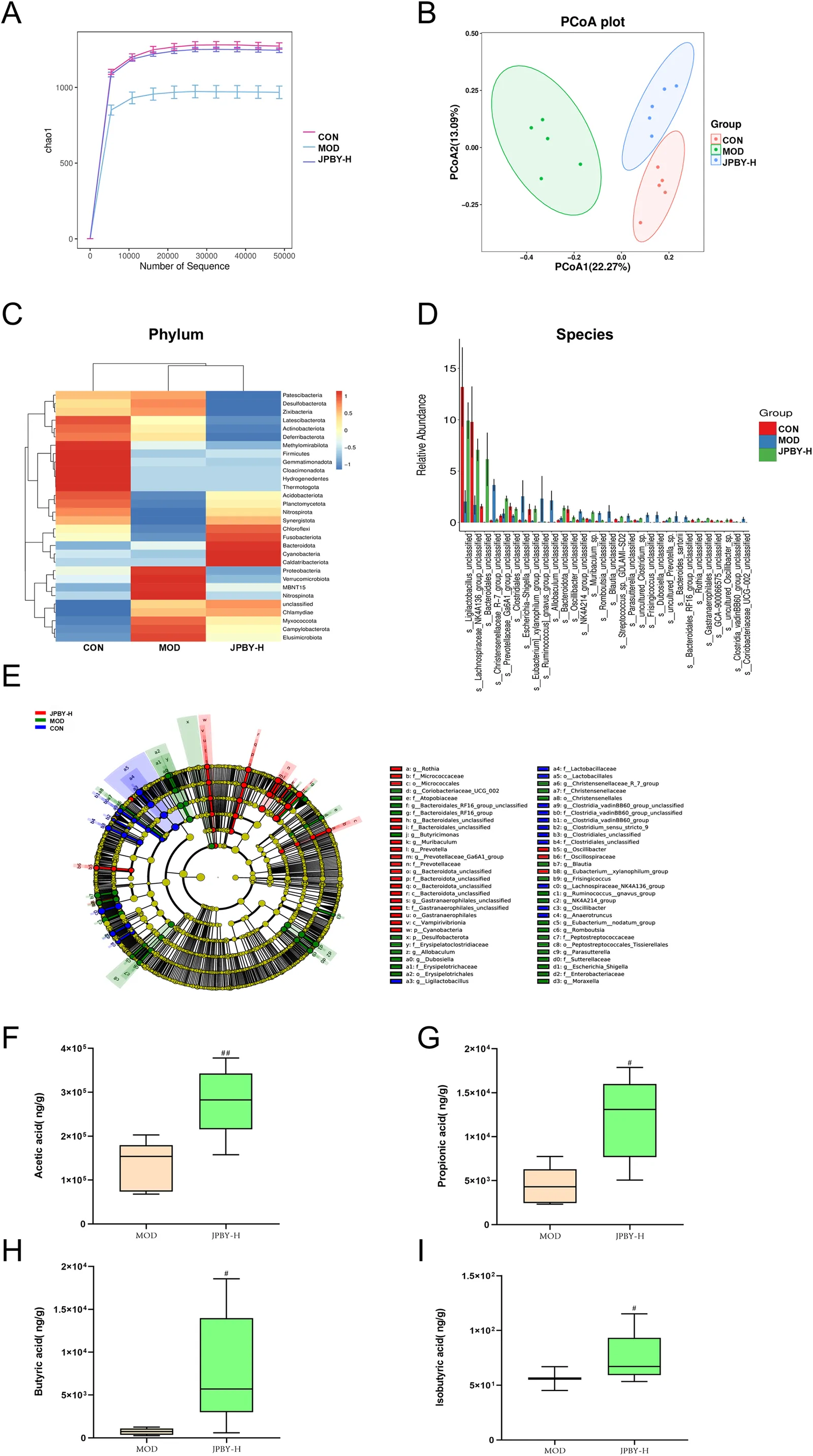
JPBY modulates the gut microbiota and SCFA metabolism in AR rats with spleen deficiency. **(A)** Alpha diversity indices (Chao1) of the gut microbiota from the CON, MOD, and JPBY-H groups. **(B)** PCoA Analysis. **(C)** Heatmap showing variations in relative abundance of microbial communities across groups at the phylum level. **(D)** Species showing significant differences among the three groups are listed by species. **(E)** LEfSe analysis. **(F–I)** Changes in the levels of acetic acid, propionic acid, butyric acid, and isobutyric acid in the intestinal contents of rats in each group.

### 3.4 Transcriptomic analysis reveals that the TLR4/NF-κB signaling pathway may be the underlying link between gut alterations and nasal inflammation

To further investigate the potential molecular mechanisms underlying the therapeutic effects of JPBY on spleen deficiency-associated AR, transcriptomic sequencing was performed on nasal mucosal tissues from the CON, MOD, and JPBY-H groups. Global transcriptomic profiling revealed extensive alterations in gene expression in the MOD group compared with the CON group, with a total of 1,081 DEGs, including 808 upregulated and 273 downregulated genes. Following JPBY treatment, 640 DEGs were identified in the JPBY-H group compared with the MOD group, comprising 176 upregulated and 464 downregulated genes. Integrated analysis of the three groups identified 398 key DEGs, indicating that JPBY markedly reshaped the aberrant transcriptional profile of the nasal mucosa in AR model rats (**Figures 4A–D**). Further analysis of the key DEGs revealed that the expression levels of TLR4, NFKB2, and IL6 were markedly increased in the MOD group compared with the CON group, whereas their expression patterns were reversed following JPBY treatment, suggesting that JPBY may counteract the aberrant activation of TLR4/NF-κB-associated inflammatory responses in AR (**Figure 4E**). To further characterize the biological functions and signaling pathways associated with these transcriptional alterations, KEGG pathway enrichment analysis was performed. The results showed that the DEGs were significantly enriched in the Toll-like receptor signaling pathway and NF-κB signaling pathway, both of which are closely involved in innate immune responses and inflammation (**Figure 4F**). Consistently, GSEA demonstrated significant enrichment of TLR- and NF-κB-related gene sets in the MOD group, whereas their enrichment was attenuated following JPBY treatment. These findings suggest that the TLR/NF-κB-associated inflammatory transcriptional program is aberrantly activated in spleen deficiency-associated AR and can be partially reversed by JPBY intervention (**Figures 4G, H**). Collectively, the transcriptomic data suggest that JPBY may alleviate spleen deficiency-associated AR by modulating the TLR4/NF-κB signaling axis and thereby suppressing aberrant inflammatory responses in the nasal mucosa.

**Figure 4:**
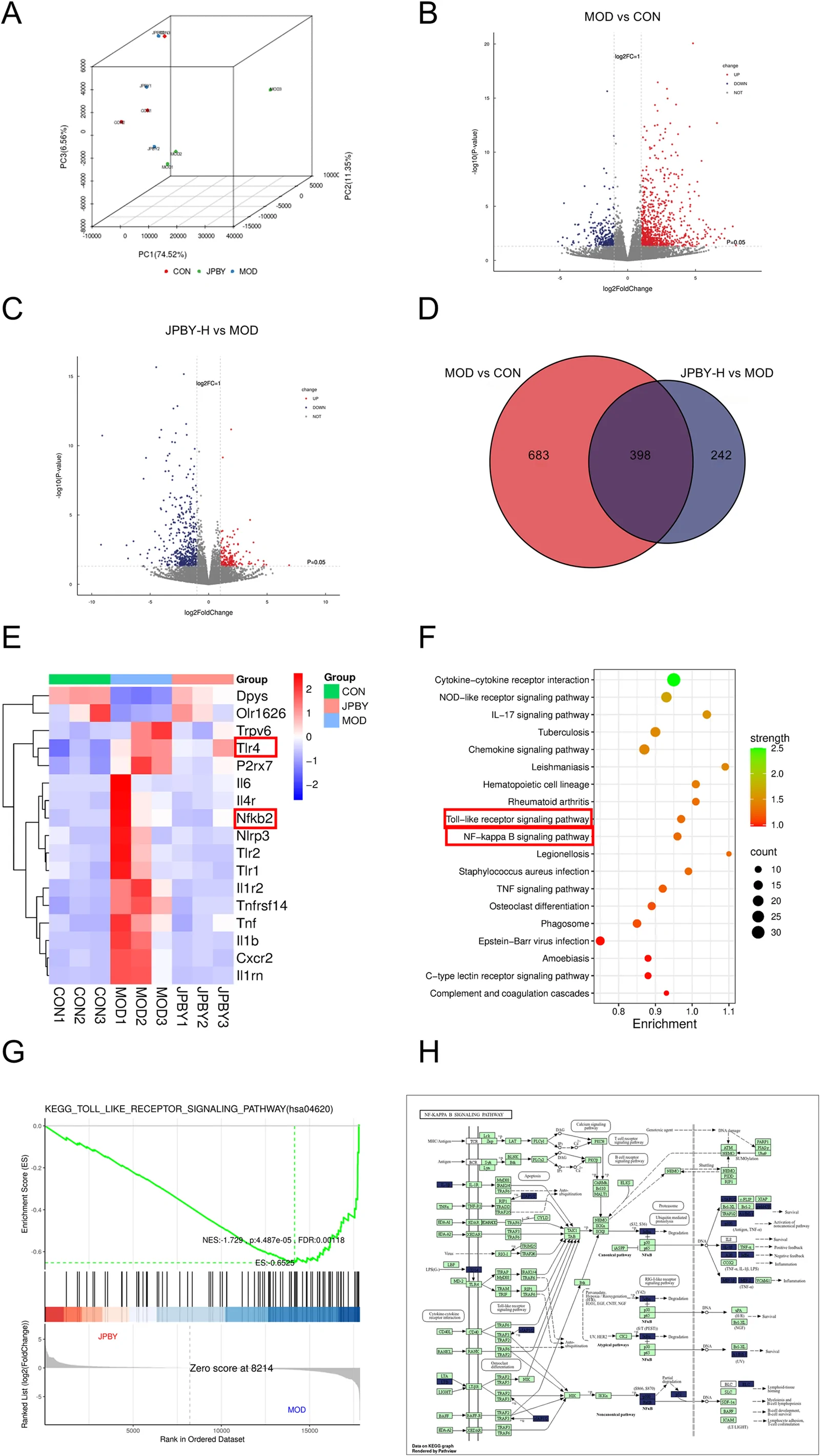
Transcriptomic analysis reveals that the TLR4/NF-κB signaling pathway may be the underlying link between gut alterations and nasal inflammation. **(A)** PCA plot of the three groups. **(B)** Volcano plots of DEGs between the MOD group and the CON group. **(C)** Volcano plots of DEGs between the JPBY-H group and the MOD group. **(D)** Venn diagram analysis of DEGs across the three groups. **(E)** Heatmap of hierarchical cluster analysis of DEGs. **(F)** KEGG analysis. **(G)** GSEA enrichment analysis. **(H)** The effects of JPBY on the NF-KAPPA B signaling pathway.

### 3.5 JPBY attenuates TLR4/NF-κB-mediated inflammation in nasal and colon tissues and restores Th1/Th2/Treg/Th17-related cytokine balance

Building on the transcriptomic findings, we further validated the involvement of the TLR4/NF-κB signaling pathway in the nasal mucosa and colonic tissues and examined its modulation by JPBY. Immunofluorescence double-labeling analysis showed that JPBY markedly attenuated the increased expression of TLR4 and MyD88 in the nasal mucosa of rats with spleen deficiency-associated AR (**Figure 5A**). Specifically, the relative fluorescence intensities of TLR4 and MyD88 were significantly lower in the JPBY-H group than in the MOD group (both *P<0.01*; **Figures 5B, C**). Consistently, Western blot analysis demonstrated that JPBY-H significantly reduced the protein levels of TLR4 and MyD88, as well as the phosphorylation level of NF-κB p65, in both colonic and nasal mucosal tissues (*P<0.05 or P<0.01*; **Figures 5D–G**, **6A–D**). To further evaluate the effects of JPBY on the systemic inflammatory response, serum levels of inflammatory cytokines were measured by ELISA. Compared with the MOD group, JPBY-H treatment significantly decreased the serum levels of IL-6, TNF-α, IL-1β, and IL-17 (*P<0.05 or P<0.01*), while significantly increasing the levels of IFN-γ and IL-10 (*P<0.01*; **Figures 6E–J**). Collectively, these findings provide further evidence that JPBY suppresses activation of the TLR4/NF-κB signaling pathway in both nasal mucosal and colonic tissues and ameliorates the associated inflammatory response. The concomitant modulation of Th1/Th2/Treg/Th17-related cytokines further suggests that JPBY may alleviate spleen deficiency-associated AR by restoring the immune-inflammatory balance.

**Figure 5:**
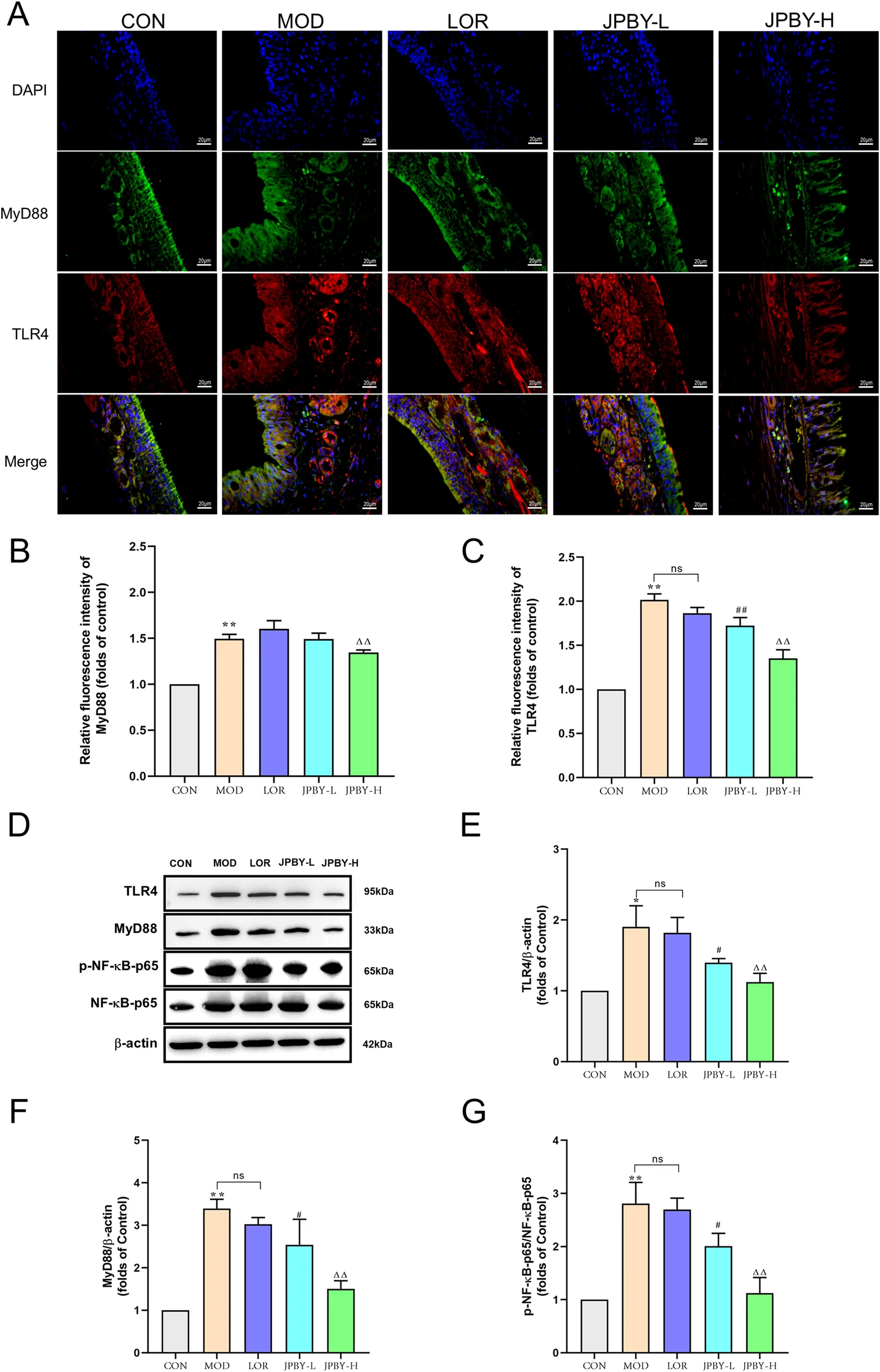
JPBY inhibited the aberrant activation of the TLR4/NF-κB signaling pathway in nasal mucosa in AR rats. **(A–C)** Immunofluorescent double-labeling of TLR4 and MyD88 in the nasal mucosa of rats in each group, along with the statistical results. **(D–G)** The expression levels of TLR4, MyD88, p-NF-κB-p65 and NF-κB-p65 proteins in the nasal mucosa of rats in each group were analyzed by Western blot (n=3). Values are presented as the mean ± SD. **p<0.01, *p<0.05, vs. the CON group. *^##^p<0.01*, *^#^p<0.05* vs. the MOD group. *^ΔΔ^p<0.01*, *^Δ^p<0.05* vs. the LOR group.

**Figure 6:**
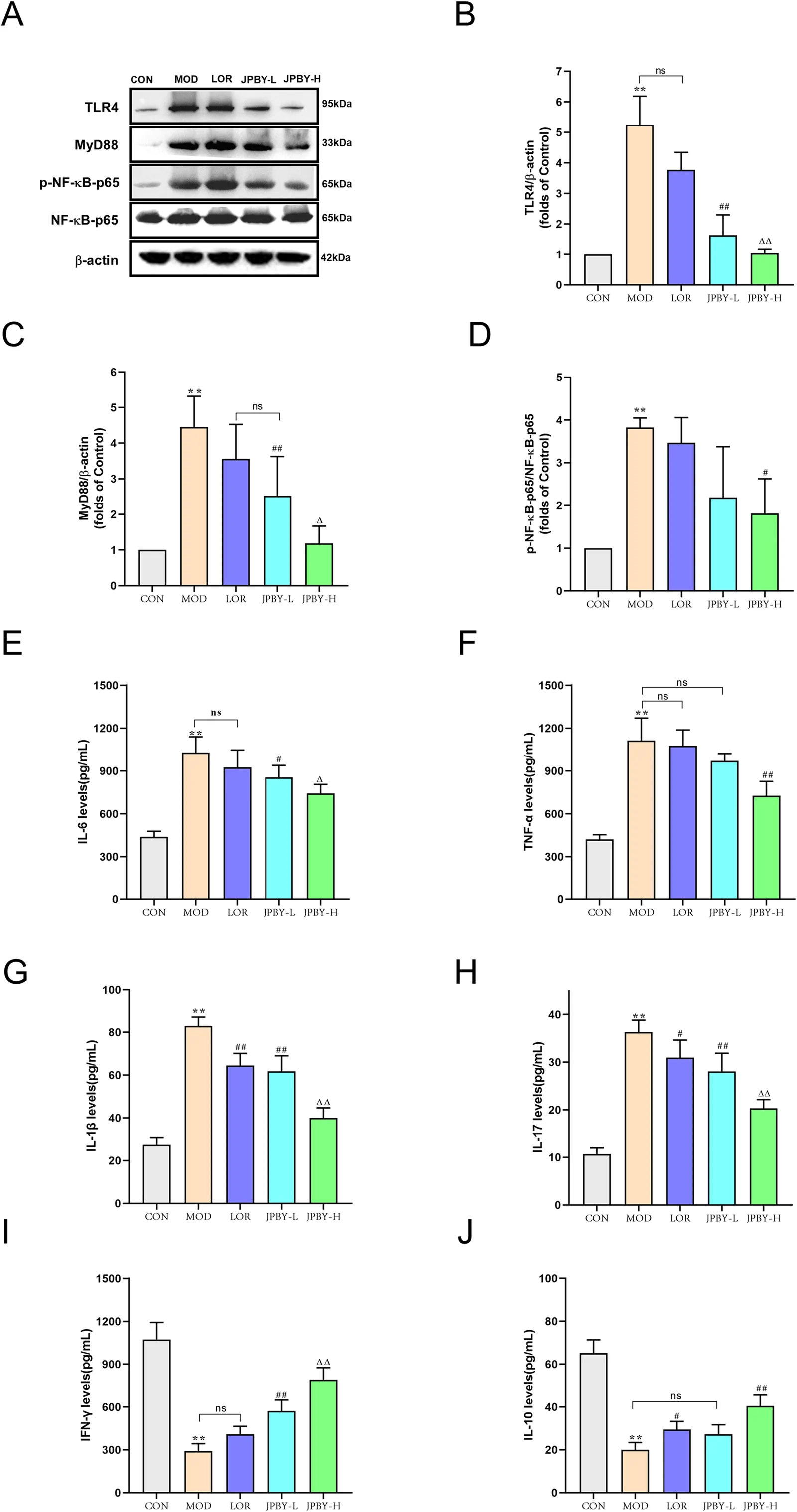
JPBY attenuates TLR4/NF-κB-mediated inflammation in colon tissues and restores Th1/Th2/Treg/Th17-related cytokine balance. **(A–D)** The expression levels of TLR4, MyD88, p-NF-κB-p65 and NF-κB-p65 proteins in the colonic tissues of rats in each group were analyzed by Western blot (n=3). **(E–J)** Serum IL-6, TNF-α, IL-1β, IL-17, IFN-γ, IL-10 levels were measured by ELISA. Values are presented as the mean ± SD. **p<0.01, *p<0.05, vs. the CON group. *^##^p<0.01*, *^#^p<0.05* vs. the MOD group. *^ΔΔ^p<0.01*, *^Δ^p<0.05* vs. the LOR group.

### 3.6 Chemical and pharmacological evidence supports a potential role of TLR4/NF-κB-related targets in the effects of JPBY

Although the *in vivo* experiments demonstrated that JPBY ameliorates both nasal mucosal injury and gut microbiota dysbiosis in AR rats, the pharmacological basis underlying these effects remains unclear. To identify the potential bioactive constituents of JPBY, the major prototype compounds entering the circulation after JPBY administration were characterized using UHPLC–MS/MS. The total ion chromatograms of JPBY, control serum, and JPBYDS are presented in **Figures 7A–C**. Using a mass-to-charge ratio (m/z) matching error of ≤1 ppm as the identification criterion, a total of 243 prototype compounds derived from JPBY were detected in JPBYDS (**Figure 7D**). Following deduplication and data quality control, 33 potential bioactive compounds were ultimately identified (**Table 2**), and 383 putative targets were subsequently predicted for these compounds. Based on these data, an active compound–target interaction network for JPBY was constructed (**Figure 7E**). To further identify the key targets potentially involved in the pharmacological effects of JPBY, a PPI network was generated using the top 90 targets ranked according to their degree values among the 383 predicted targets (**Figure 7F**). Among these, IL6, ESR1, NFKB1, JUN, and IL2 exhibited relatively high connectivity and were identified as potential hub targets for further investigation. KEGG pathway enrichment analysis of the JPBY compound–target network further revealed that the identified bioactive compounds were associated with multiple signaling pathways involved in diverse biological processes (**Figure 7G**). Collectively, these findings indicate that JPBY contains multiple bioactive constituents capable of interacting with a broad range of protein targets, suggesting a multi-component, multi-target pharmacological basis for its therapeutic effects against AR.

**Figure 7:**
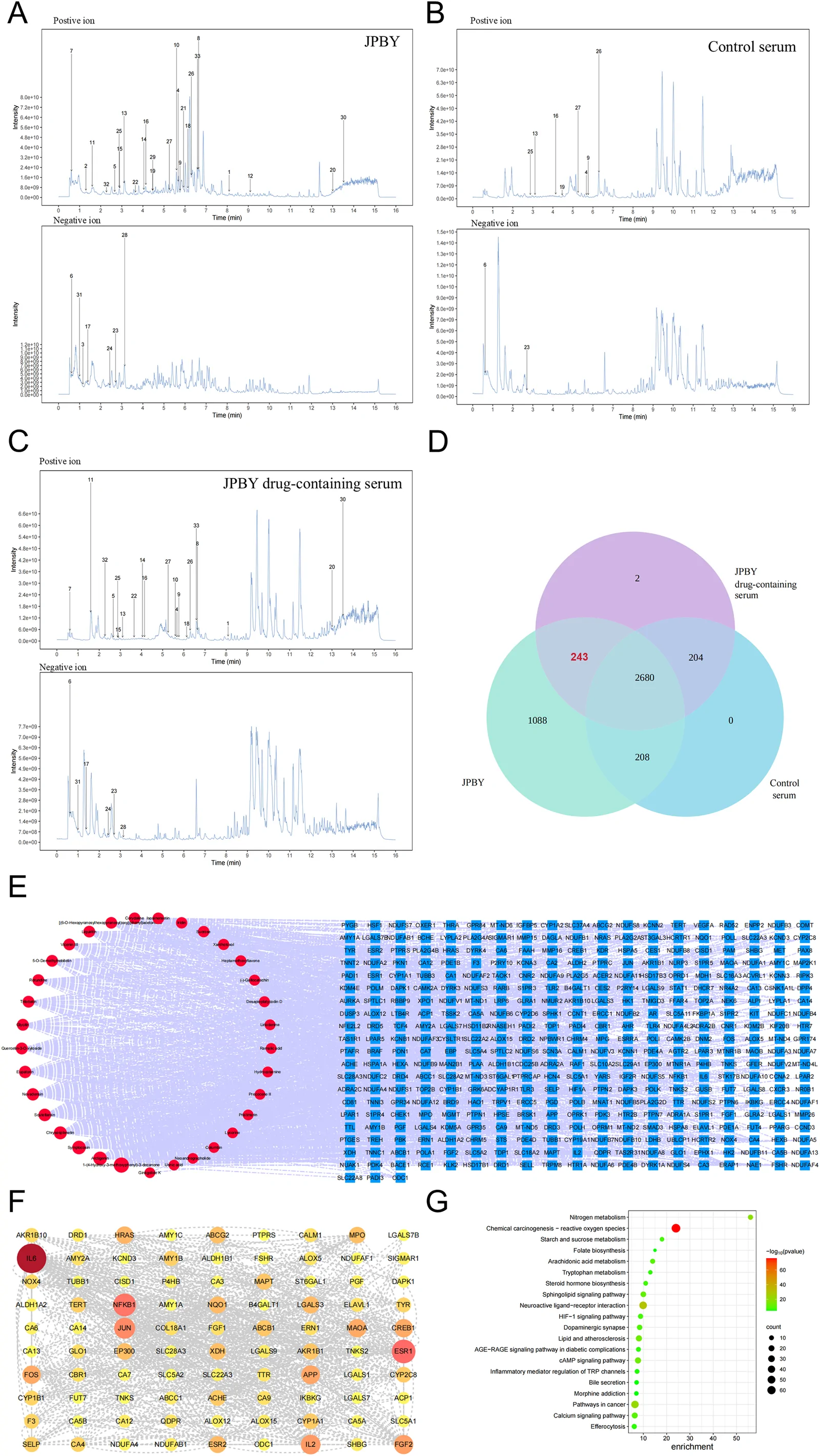
Identification of the major components of JPBY absorbed into the bloodstream. **(A)** Total ion flow diagram for component identification in JPBY samples. **(B)** Total ion mobility diagram for component identification in control serum group samples. **(C)** Total ion flow diagram for the identification of sample components in JPBY drug-containing serum group samples. **(D)** Venn diagram of cross-active components of three groups. **(E)** JPBY active ingredient compound-target network. **(F)** PPI network constructed by extracting the top 90 targets based on degree values. **(G)** The top 20 enriched KEGG pathways.

**Table 2:** Chemical components of JPBY drug-containing serum.

No.	Name	Formula	Reference Ion	RT (sec)	m/z
1	Arctigenin	C_21_H_24_O_6_	+	485.471	395.1469991
2	[(6-O-Hexopyranosylhexopyranosyl)oxy](phenyl)acetonitrile	C_20_H_27_NO_11_	+	77.8468	480.1477
3	Trilobatin	C_21_H_24_O_10_	−	69.348	435.1301699
4	5-O-Demethylnobiletin	C_20_H_20_O_8_	+	340.402	389.1228855
5	Xanthotoxol	C_11_H_6_O_4_	+	159.843	203.0340731
6	Sucrose	C_12_H_22_O_11_	−	37.1341	341.1087625
7	Hydrocotarnine	C_12_H_15_NO_3_	+	36.88665	222.1119241
8	Heptamethoxyflavone	C_22_H_24_O_9_	+	398.492	433.1480676
9	Isosinensetin	C_20_H_20_O_7_	+	345.833	395.1091822
10	Eupatorin	C_18_H_16_O_7_	+	335.814	345.0967299
11	Liquiritin	C_21_H_22_O_9_	+	95.7958	419.1338221
12	Precocene II	C_13_H_16_O_3_	+	545.5625	221.1170388
13	Liriodenine	C_17_H_9_NO_3_	+	186.406	276.0650043
14	Nevadensin	C_18_H_16_O_7_	+	242.638	345.0966965
15	Glycitin	C_22_H_22_O_10_	+	173.802	447.1279332
16	Desapioplatycodin D	C_52_H_84_O_24_	+	248.444	1093.540139
17	Vicenin III	C_26_H_28_O_14_	−	83.26835	563.1403992
18	1-(4-Hydroxy-3-methoxyphenyl)-3-decanone	C_17_H_26_O_3_	+	368.097	261.184798
19	Lycorine	C_16_H_17_NO_4_	+	267.671	288.1230102
20	Pristimerin	C_30_H_40_O_4_	+	780.299	465.2971535
21	Usnic acid	C_18_H_16_O_7_	+	355.717	345.0966575
22	Chrysosplenetin	C_19_H_18_O_8_	+	218.929	375.1072672
23	Iridin	C_24_H_26_O_13_	−	162.276	521.1305574
24	(-)-Gallocatechin	C_15_H_14_O_7_	−	145.748	305.0671069
25	Corydaline	C_22_H_27_NO_4_	+	172.447	370.2010302
26	Ramalic acid	C_18_H_18_O_7_	+	377.809	347.1118432
27	Rotundine	C_21_H_25_NO_4_	+	315.423	356.1829904
28	Scutellarein	C_15_H_10_O_6_	−	188.457	285.0401266
29	Ginkgolide K	C_20_H_22_O_9_	+	268.059	407.1339108
30	Neoandrographolide	C_26_H_40_O_8_	+	811.1695	481.2834737
31	Quercetin-3-O-xyloside	C_20_H_18_O_11_	−	59.7709	433.0812908
32	Symplocosin	C_26_H_32_O_11_	+	136.39	538.2289977
33	Columbin	C_20_H_22_O_6_	+	395.4735	359.1488286

To further investigate the potential mechanisms underlying the effects of JPBY on AR in rats with spleen deficiency, we used network pharmacology to predict key targets and constructed a comprehensive network, with particular attention to pathways potentially associated with gut–nose axis-related processes. The results showed that after systematically screening target genes across multiple databases and identifying overlapping genes via Venn diagram analysis, 13 genes were ultimately identified as key genes (**Figure 8A**). Subsequently, the key gene targets were imported into the STRING database to construct a PPI network, revealing that IL6, TLR4, and NFKB1 are all key targets (**Figure 8B**). GO and KEGG analyses (**Figures 8C, D**) indicated that these key genes are involved in biological processes such as oxidative stress responses, immune response activation, cell surface receptor signaling pathways, and the binding and function of transcriptional co-regulators. In addition, the Toll-like receptor signaling pathway and the Th1 and Th2 cell differentiation signaling pathways were both significantly enriched. Subsequently, a composite network was constructed based on key gene targets, their associated compounds, and gut microbiota (**Figure 8E**). This further identified 22 active components of JPBY targeting key gene targets and 9 key gut microbiota species, which collectively play a significant role in the treatment of AR with JPBY. TLR4, MyD88, NF-κB, and IL-6 were selected as representative receptor proteins, with xanthotoxol and arctigenin serving as ligands for molecular docking analysis. The binding modes and protein–ligand interactions were evaluated and visualized (**Figure 9**). Both xanthotoxol and arctigenin showed potential binding interactions with TLR4, MyD88, NF-κB, and IL-6, with hydrogen bonds and hydrophobic interactions contributing to the predicted binding. The corresponding pKi values were further calculated to characterize the predicted binding affinities of these protein–ligand complexes (**Table 3**). These complementary docking and pKi analyses provide additional computational evidence for the potential interactions of xanthotoxol and arctigenin with multiple key inflammatory targets.

**Figure 8:**
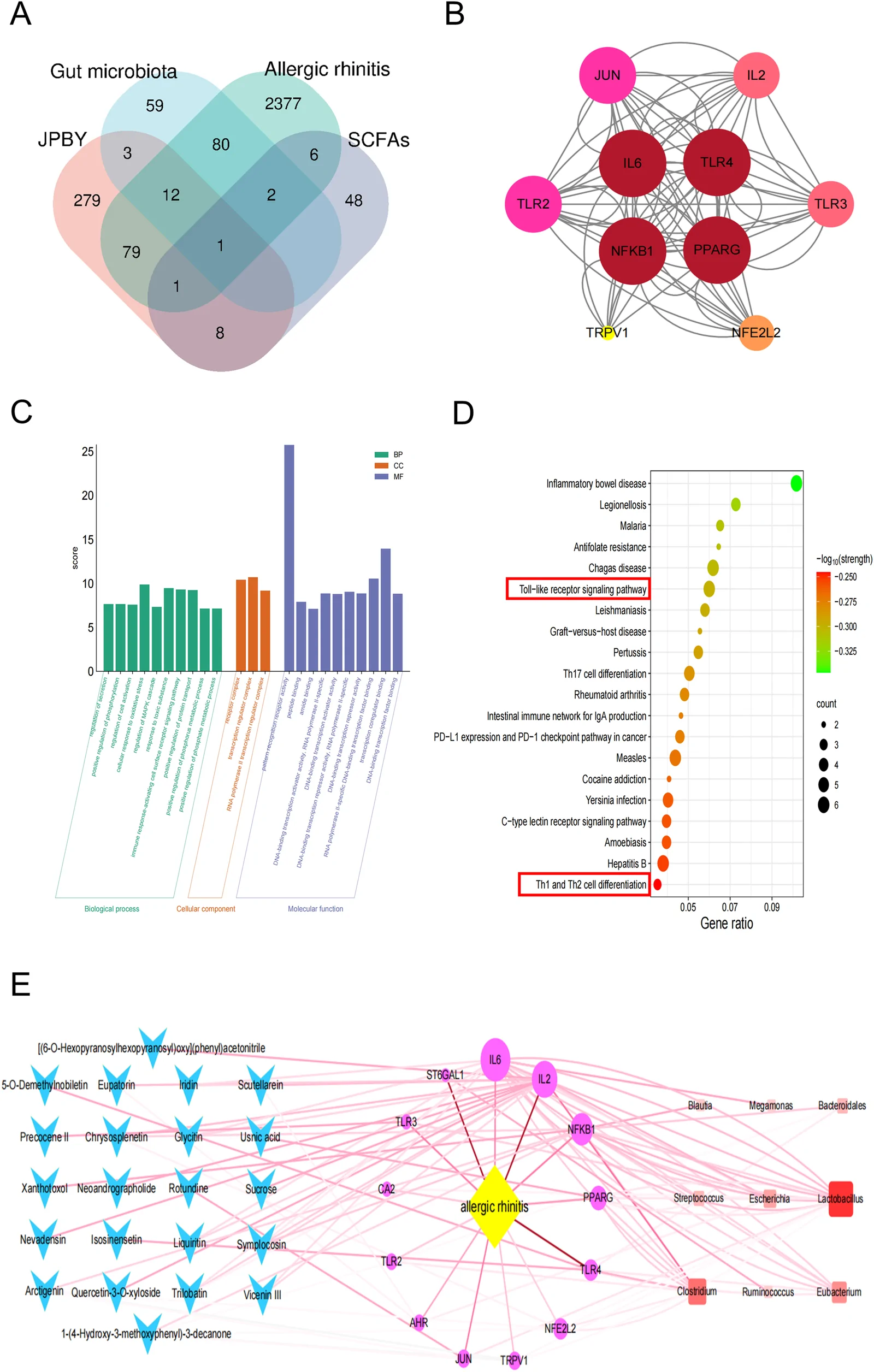
Chemical and pharmacological evidence supports that TLR4/NF-κB-related targets are involved in the mechanism by which JPBY modulates the gut-nose axis. **(A)** Venn analysis. **(B)** PPI network of key genes **(C, D)** GO and KEGG analysis for key genes. **(E)** A composite network of key gene targets and their associated compounds and gut microbes was constructed.

**Figure 9:**
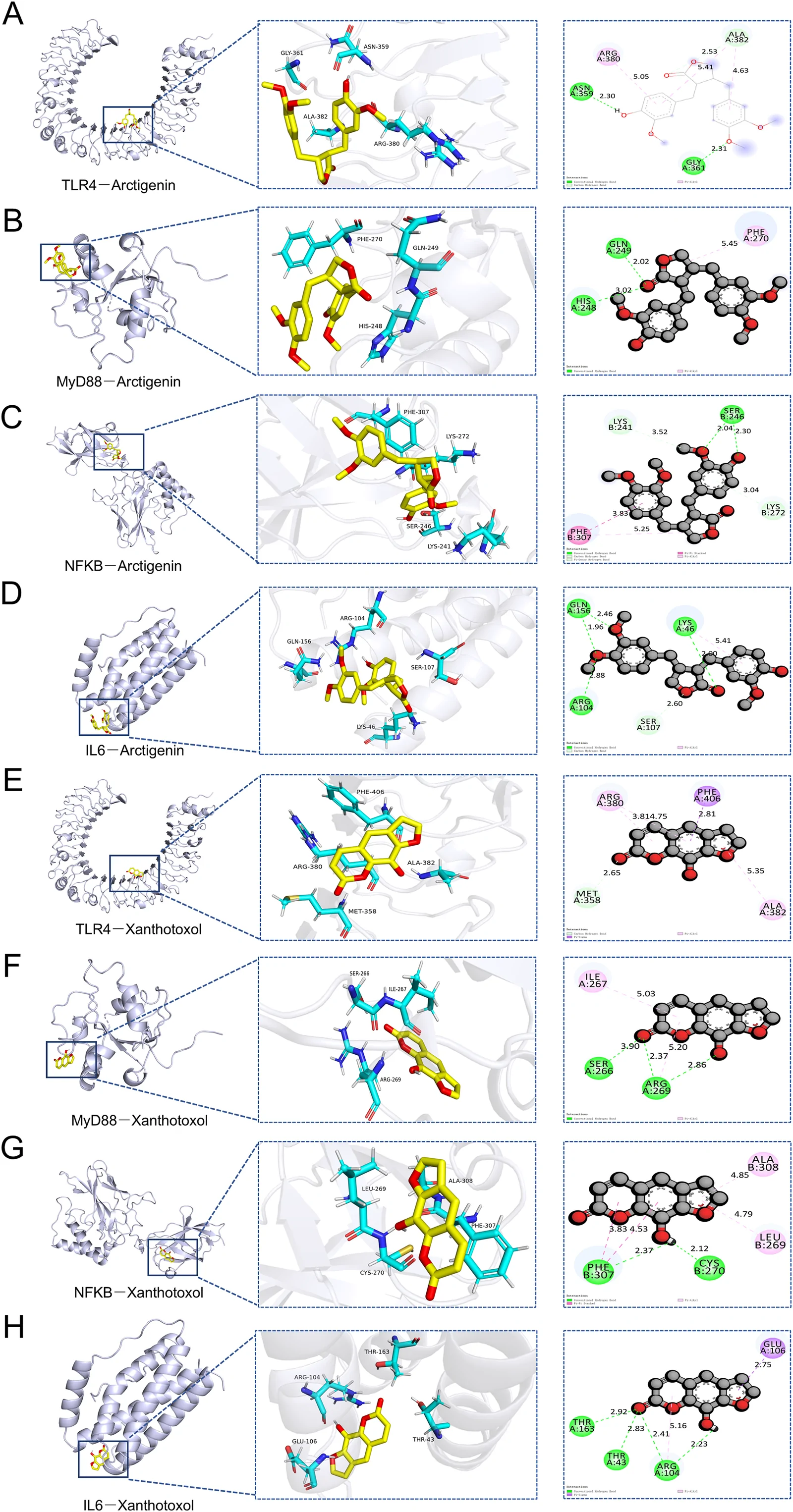
Molecular docking. **(A–D)** Molecular docking analysis and visualization of arctigenin binding to TLR4, MyD88, NF-κB, and IL-6. **(E–H)** Molecular docking analysis and visualization of xanthotoxol binding to TLR4, MyD88, NF-κB, and IL-6.

**Table 3:** The docking scores.

Protein	Docking score (kcal/mol)	
	Xanthotoxol	Arctigenin
TLR4	-6.0	-5.7
MyD88	-6.1	-5.7
NFKB	-5.9	-6.5
IL-6	-6.3	-6.0

## 4 Discussion

AR is a chronic and recurrent inflammatory disorder for which current therapies primarily focus on symptom control and long-term disease management (1). Increasing recognition of distinct clinical phenotypes and TCM syndrome characteristics has highlighted the potential value of individualized therapeutic strategies (8). Spleen deficiency syndrome is commonly observed in patients with AR (32), and the emerging concept of the gut–nose axis provides a complementary framework for understanding how intestinal microbial homeostasis, mucosal barrier function, and immune-inflammatory responses may interact in this phenotype (33–35). Against this background, our study suggests that JPBY may exert its therapeutic effects on spleen-deficiency AR through coordinated regulation of the gut–nose axis. Rather than acting solely through a conventional anti-inflammatory mechanism, JPBY appeared to influence multiple interconnected components of this axis, with TLR4/NF-κB signaling emerging as a potential molecular link between microbial–metabolic alterations and immune regulation. These findings extend the growing evidence that the gut–nose axis contributes to allergic inflammation and provide a potential mechanistic framework for understanding the pharmacological effects of JPBY in spleen-deficiency AR. The following sections discuss how JPBY may modulate gut microbiota and microbial metabolites, preserve mucosal barrier integrity, and regulate TLR4/NF-κB-associated immune responses, as well as the potential contribution of its bioactive constituents to these effects.

This study established a combined model of AR with spleen deficiency syndrome in rats using OVA sensitization combined with bitter-cold purgatives and irregular eating patterns. OVA sensitization induces typical IgE-mediated nasal allergic reactions, while interventions such as bitter-cold purgatives and irregular eating patterns were used to simulate the pathological state of “impaired spleen transport and transformation” in TCM. Previous studies have also shown that combining allergen sensitization with interventions related to TCM syndromes can establish animal models exhibiting both the immunological characteristics of AR and the corresponding syndrome phenotypes (36). Compared with simple AR models, the syndrome-disease integrated model can simultaneously present both allergic inflammation and spleen-deficiency-related phenotypes within the same experimental system, thereby providing an experimental foundation for studying the mechanism of action of JPBY from the perspective of integrating modern disease with TCM syndromes (37). In addition to behavioral changes, this study found that rats with spleen-deficiency-type AR also exhibited significant abnormalities in gastrointestinal hormone secretion and impaired digestive function, suggesting that gastrointestinal dysfunction may be involved in the pathophysiological process of spleen-deficiency-type AR. Previous studies have shown that gastrointestinal digestive function may play a role in the regulation of allergic reactions. Reduced gastric digestive capacity can increase the entry of intact allergens into the intestine and induce immunological sensitization, whereas supplementation with gastric digestive enzymes and restoration of a normal gastric digestive environment can alleviate experimental food allergic reactions (38). This study found that the JPBY intervention not only improved nasal and spleen-deficiency-related symptoms in rats with AR but also reduced serum OVA-sIgE levels and improved gastrointestinal digestive function. Therefore, the anti-AR effects of JPBY may involve not only the regulation of local nasal inflammation but also improvements in gastrointestinal function and gastrointestinal immune homeostasis. However, whether there is a direct causal relationship between improved gastrointestinal digestive function and the reduction in AR symptoms and OVA-sIgE levels requires further verification through studies of the intestinal barrier, gut microbiota, and relevant immune pathways. Furthermore, histopathological staining revealed that in the spleen deficiency-type AR rat model, in addition to classic inflammatory damage in the nasal mucosa, the intestinal mucosa also exhibited significant inflammatory cell infiltration and loss of intestinal crypt structure. This is consistent with the pathological manifestations of spleen deficiency syndrome reported in previous studies (39, 40). Following treatment with JPBY, pathological damage to both the nasal and intestinal mucosa showed some improvement, and the integrity of the mucosal barrier was significantly enhanced. In recent years, epithelial barrier dysfunction has been identified as a key factor in the onset and progression of AR (41). As the first line of defense in the respiratory tract against external harmful substances, the integrity of the nasal epithelial barrier is crucial for maintaining the homeostasis of the nasal environment. In AR patients, disruption of the epithelial barrier not only facilitates the penetration of allergens through the mucosa but also triggers a series of immune-inflammatory cascade reactions; significantly reduced expression of ZO-1, E-cadherin, and occludin is closely associated with the severity of AR (42).

In addition, this study further utilized 16S rRNA sequencing technology to investigate the regulatory effects of JPBY on the composition of the gut microbiota and, in combination with metabolomic analysis, assessed the changes in SCFAs. The results showed that JPBY intervention significantly improved gut microbiota dysbiosis in AR rats with spleen deficiency, reshaped the microbial community structure, and promoted the production of various SCFAs with immunomodulatory functions. Following high-dose JPBY treatment, the relative abundance of Ligilactobacillus and Lachnospiraceae increased significantly, while levels of acetic acid, propionic acid, butyric acid, and isobutyric acid rose markedly, suggesting that JPBY may exert anti-inflammatory and immunoregulatory effects by modulating the “gut microbiota–metabolite–immune response” axis. Recent studies have shown that SCFAs derived from the gut microbiota are not only important end products of microbial metabolism but also key signaling molecules linking the gut microbiome to the host immune system (43). Specifically, acetic acid-one of the most abundant SCFAs in the gut-can enhance intestinal epithelial barrier function and inhibit TLR4 expression and downstream NF-κB activation, thereby reducing the release of pro-inflammatory factors such as TNF-α, IL-6, and IL-1β (44). Propionic acid, on the other hand, can alleviate abnormal immune activation by reducing the nuclear translocation of the NF-κB p65 subunit and thereby inhibiting the transcription of inflammation-related genes. Furthermore, propionic acid has been reported to suppress dendritic cell-driven Th2-type immune responses, which holds potential significance for correcting Th1/Th2 immune imbalances in allergic diseases (44, 45). As an important metabolite produced by dominant bacterial communities such as the Lachnospiraceae, butyric acid plays a key role in maintaining intestinal immune homeostasis. Previous studies have shown that butyric acid promotes the expression of anti-inflammatory genes and enhances the expression of tight junction proteins, thereby improving intestinal barrier integrity and further inhibiting the activation of the TLR4/MyD88/NF-κB inflammatory signaling pathway (46, 47). As an important member of probiotics, Ligilactobacillus demonstrates significant potential in the treatment and management of AR. Studies have shown that Ligilactobacillus can effectively promote Th1-type immune responses and suppress Th2-type responses by regulating the cytokine profile, thereby effectively alleviating AR (48). Therefore, this study found that the mechanism by which JPBY improves AR may not only rely on the direct regulation of immune and inflammatory responses but also involve a systemic remodeling of the gut microbiota’s composition and metabolic functions.

To further clarify the pharmacological basis and potential mechanisms of action of JPBY in the treatment of AR, this study first identified the active components of JPBY that enter the body following oral administration using serum pharmacochemistry. Combining network pharmacology, molecular docking, and multi-omics analysis, the study constructed a multi-level action network comprising “key components, key gut microbiota, and key targets.” Previous studies have shown that the gut microbiota can significantly influence the bioavailability, metabolic transformation, and *in vivo* efficacy of active components in TCM; therefore, the combination of serum pharmacochemistry and gut microbiota analysis helps provide a more comprehensive explanation of the pharmacological basis of JPBY’s *in vivo* efficacy (49). Based on the analysis of the composite network, among the components of JPBY that enter the bloodstream, xanthotoxol and arctigenin may be key candidate components linking JPBY’s “direct anti-inflammatory effects” and “gut microbiota-modulating effects.” Molecular docking results showed that xanthotoxol and arctigenin exhibit strong binding affinity for IL-6 and TLR4, suggesting that they may be directly involved in the regulation of inflammatory signaling. Previous studies have shown that xanthotoxol possesses significant anti-inflammatory and immunomodulatory activity. It exerts its anti-inflammatory effects by inhibiting IκBα phosphorylation and NF-κB p65 nuclear translocation, thereby reducing the expression of pro-inflammatory mediators such as IL-6 and IL-1β (50). Furthermore, a TCM extract containing xanthotoxol significantly reduced serum IgE, histamine, and Th2-related inflammatory cytokine levels in an OVA-induced AR model and inhibited NF-κB signaling activation in mast cells (51). Arctigenin also exhibits strong anti-inflammatory and anti-allergic activity, inhibiting mast cell-mediated allergic reactions and the activity of various pro-inflammatory enzymes (52). Notably, this study observed that the high-dose group of JPBY demonstrated a more pronounced therapeutic effect than the low-dose group, suggesting that JPBY may exhibit a dose-exposure-effect relationship. For multi-component TCM formulations, their dose-dependent pharmacological effects may be related to the combined exposure to and synergistic action of various active ingredients and their gut microbiota metabolites (53). Previous studies have shown that the gut microbiota can participate in the biotransformation of active ingredients in TCM, thereby further influencing their bioavailability and pharmacological activity (54). Therefore, the more pronounced pharmacological effects observed with high doses of JPBY in this study may be related to increased effective exposure to key active ingredients and their gut microbial metabolites, as well as the resulting pharmacokinetic and pharmacodynamic synergistic effects (55). This mechanism may constitute an important *in vivo* basis for the dose-dependent effects of JPBY.

Compared with the positive control drug loratadine, JPBY may exhibit a different pharmacological mechanism of action. As a second-generation H1 receptor antagonist, loratadine primarily alleviates AR symptoms by blocking histamine–H1 receptor signaling (1). While loratadine primarily acts on the symptomatic effects mediated by histamine, JPBY may regulate AR inflammation at multiple levels through holistic modulation of inflammation and immune homeostasis. Therefore, within the context of Western medical treatment, the potential advantage of JPBY lies not merely in replacing H1 receptor antagonists, but rather in further intervening in the inflammatory and immune imbalances of AR through the synergistic regulation of its multi-component, multi-target nature and the gut-immune axis, thereby providing a complementary mechanism and a potential combination therapy strategy for traditional antihistamine treatment. Regarding the mechanism of action, KEGG enrichment analysis, transcriptomics, and animal experiments collectively point to the TLR signaling pathway and the Th1/Th2 cell differentiation pathway, further suggesting that TLR4/NF-κB may serve as a key hub linking the active components of JPBY, the gut microbiota, and the AR inflammatory response. As a key pattern recognition receptor in the innate immune system, excessive activation of TLR4 promotes NF-κB activation through MyD88-dependent signaling, which in turn induces the release of IL-6, TNF-α, and other pro-inflammatory mediators, thereby contributing to local inflammation and immune dysregulation in AR (56). Notably, JPBY treatment was associated with reduced TLR4/NF-κB-associated signaling in both the nasal mucosa and colon, indicating that its effects may extend beyond the local regulation of nasal inflammation. The coordinated changes observed in the intestinal and nasal compartments suggest that JPBY may influence inflammatory responses at both sites, potentially involving gut–nose axis-related processes (**Figure 10**). These findings provide a possible mechanistic framework for understanding the effects of JPBY on spleen-deficiency-type AR; however, whether the coordinated intestinal and nasal responses are causally connected through the gut–nose axis remains to be determined. Future studies could employ causal validation strategies, including fecal microbiota transplantation (FMT), specific bacterial strain interventions, SCFA supplementation or receptor blockade, and targeted modulation of intestinal or nasal barrier function, to further clarify the temporal sequence and causal relationships among the gut microbiota, SCFAs, mucosal barrier function, and nasal inflammation.

**Figure 10:**
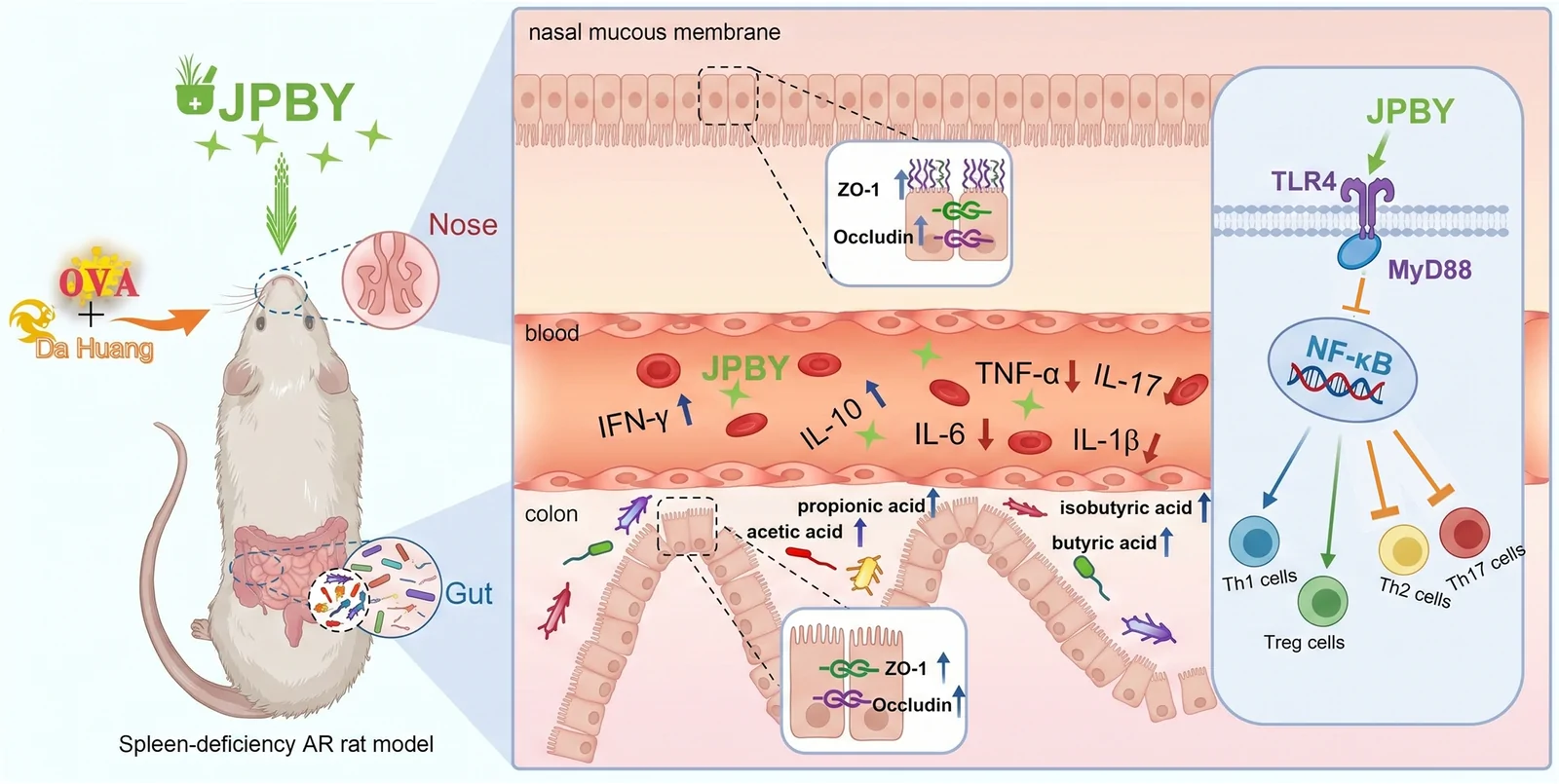
The molecular mechanism of action. The mechanism of action of JPBY may be related to inhibition of the TLR4/NF-κB signaling pathway in the gut-nose axis, repair of the mucosal barrier and correction of the Th1/Th2/Treg/Th17-related cytokine imbalance.

## 5 Limitations

This study has several limitations. First, although JPBY-associated changes in gut microbiota, SCFAs, mucosal barrier function, immunity, and inflammation were observed in parallel with improvements in AR-related phenotypes, the present study did not establish their causal relationships. Future studies using FMT, microbiota depletion, or targeted interventions against key microbial taxa or metabolites are warranted to clarify the causal role of gut–nose interactions. Second, the dose–response relationships of the active components of JPBY remain unclear, and further systematic multi-omics and target-validation studies are needed to identify the key active components and molecular pathways. Third, only male rats were used, which may limit the generalizability of the findings; future studies should investigate potential sex-related differences in AR pathogenesis and therapeutic responses. Overall, the findings suggest a potential involvement of gut–nose axis-related mechanisms in the effects of JPBY on AR, but further causal and translational studies are required to validate this proposed mechanism and its clinical significance.

## 6 Conclusions

Overall, this study provides preliminary evidence for the potential involvement of gut–nose axis-related processes in spleen-deficiency-type AR. JPBY treatment was associated with coordinated changes in gut microbiota, SCFA metabolism, intestinal and nasal mucosal barrier function, and TLR4/NF-κB-associated inflammatory responses. These findings provide experimental support for understanding spleen-deficiency-type AR from the TCM perspective of “treating nasal disorders by regulating the gut” and suggest the potential value of gut–nose axis-related pathways as targets for further investigation of AR and related TCM formulations.

## Data Availability

The datasets presented in this study can be found in online repositories. The names of the repository/repositories and accession number(s) can be found in the article/**Supplementary Material**.
